# Use of an oxygen planar optode to assess the effect of high velocity microsprays on oxygen penetration in a human dental biofilms in-vitro

**DOI:** 10.1186/s12903-020-01217-0

**Published:** 2020-08-21

**Authors:** Yalda Khosravi, Raja Durga Prasad Kandukuri, Sara Palmer, Erin S. Gloag, Sergey M. Borisov, E. Michelle Starke, Marilyn T. Ward, Purnima Kumar, Dirk de Beer, Arjun Chennu, Paul Stoodley

**Affiliations:** 1 Department of Microbial Infection and Immunity, Ohio State University, Columbus, USA; 2 Max Planck Institute for Marine Microbiology, Bremen, Germany; 3 College of Dentistry, The Ohio State University, Columbus, OH USA; 4 Institute of Analytical Chemistry and Food Chemistry Graz University of Technology Stremayrgasse, Graz, Austria; 5 Philips Oral Healthcare, Bothell, Washington, 98021 USA; 6 Department Orthopaedics, Ohio State University, Columbus, USA; 7 National Centre for Advanced Tribology (nCATS), Mechanical Engineering, University of Southampton, Southampton, UK

**Keywords:** Mechanical disruption, Microspray, Oral, Biofilm, Planar optodes, Dissolved oxygen

## Abstract

**Background:**

Dental plaque biofilms are the causative agents of caries, gingivitis and periodontitis. Both mechanical and chemical strategies are used in routine oral hygiene strategies to reduce plaque build-up. If allowed to mature biofilms can create anoxic microenvironments leading to communities which harbor pathogenic Gram-negative anaerobes. When subjected to high velocity fluid jets and sprays biofilms can be fluidized which disrupts the biofilm structure and allows the more efficient delivery of antimicrobial agents.

**Methods:**

To investigate how such jets may disrupt anoxic niches in the biofilm, we used planar optodes to measure the dissolved oxygen (DO) concentration at the base of in-vitro biofilms grown from human saliva and dental plaque. These biofilms were subject to “shooting” treatments with a commercial high velocity microspray (HVM) device.

**Results:**

HVM treatment resulted in removal of much of the biofilm and a concurrent rapid shift from anoxic to oxic conditions at the base of the surrounding biofilm. We also assessed the impact of HVM treatment on the microbial community by tracking 7 target species by qPCR. There was a general reduction in copy numbers of the universal 16S RNA by approximately 95%, and changes of individual species in the target region ranged from approximately 1 to 4 log reductions.

**Conclusion:**

We concluded that high velocity microsprays removed a sufficient amount of biofilm to disrupt the anoxic region at the biofilm-surface interface.

## Background

Oral microbial biofilms are complex communities of bacteria and fungi which grow on the teeth and soft tissues of the mouth. Dental/plaque biofilm formation progresses along an ecological succession starting with early commensal colonizers such as facultative anaerobic oral streptococci [1–4].

In the early stages of plaque formation Streptococci*,* such as *S. oralis* and *S. gordonii*, attach, followed by a succession of organisms, which attach to the streptococci through a process known as coaggregation [5, 6]. As these organisms metabolize dietary sugars they consume oxygen to lower the local dissolved oxygen (DO) concentration at the base of the biofilm, produce acids, and metabolites that may favor or antagonize other community members. As the biofilm matures, anaerobic bacteria such as *Veillonella parvula*, *Fusobacterium nucleatum* and *Porphyromonas gingivalis* proliferate [7].

For those on a high- sugar diet acid fermentation by the cariogenic pathogen, *S. mutans* results in lowering the pH within the biofilm, as well as the production of an extracellular insoluble glucans which provides volume, structure and mechanical stability to the developing biofilm. As the thickness of the biofilm increases diffusion limitation of oxygen into the biofilm and metabolites out of the biofilm results in the build-up of steep gradients from physiological conditions to acidic and anoxic within 100 μm [8]. The oral cavity and especially the periodontal pocket provides a unique eco-system for microbial organisms and harbors a diverse microbiota with up to 700 prokaryote species [9]. One model for pathogenesis of periodontitis suggests that periodontal microbial communities can be clustered into complexes that are associated with disease severity [10–12]. Seminal work, at that time based on culture dependent techniques, of Socransky identified a “red complex” harboring *Porphyromonas gingivalis*, *Tannerella forsythia*, and *Treponema denticola*, associated with the severe form of periodontitis. Further complexes included an intermediate orange complex with, e.g., *Fusobacterium nucleatum* and a yellow and green complex dominated by *Streptococcus* spp., the latter being associated with health. Support for a classical role for the red complex as direct pathogens came from the observation by Holt showing induction of periodontitis upon oral implantation of these bacteria in non-human primates [13]. However, more recent concepts suggest that keystone pathogens can disrupt tissue homeostasis and change the composition of the commensal microbiota thereby generating host immune modulation and dysbiosis, that is responsible for periodontitis [14, 15]. Such a concept takes into account observations that periodontal pathogens often are low abundant and can be present in healthy people [16]. Periodontitis resembles the process of microbial succession with an increase of periodontitis-associated taxa while health-associated species remain but decrease in number. In turn, the microbial community structure changes significantly, and biomass typically increases [10].. *P. gingivalis* can also occur in the saliva and supragingival plaque both in healthy patients (≈10%) and those with chronic periodontitis (≈60%) [17], presumably protected in the microaerophilic or anoxic environment of attached biofilm or detached biofilm aggregates.

Dental biofilms, are recalcitrant to antimicrobial agents as well as to complete mechanical removal, when growing in places difficult to reach with tooth-brush bristles. High velocity microspray (HVM) is a new technology which provides an alternative to string flossing for interproximal plaque management, and has been shown in clinical trials to reduce plaque and gingival index when used in combination with manual and electric toothbrushes and mouthwashes [18–20]. However, the precise mechanism(s) of action are not known. In a recent in vitro study we reported that a commercial HVM device designed for interproximal cleaning removed significant amounts of a *S. mutans* biofilm, but intriguingly caused the small amount of remaining biofilm to flow, forming interfacial instabilities between the air/water spray and the biofilm [21]. This effect allowed much deeper penetration of particles and antimicrobial agents into the biofilm than could be achieved by shaking at 200 rpm for 30 s alone [22]. We hypothesized that a combination of removal and mixing might disrupt the pathogenic anoxic environment at the base of the biofilm, thus potentially directing the biofilm community from pathogenic to commensal by modifying the environment, rather than purely trying to kill the biofilm bacteria with antimicrobial agents.

To test our hypotheses biofilms grown from saliva and toothbrush recovered plaque from healthy volunteers were grown on oxygen planar optodes [23, 24]. The oxygen concentration was measured prior to and after two daily shootings with a high velocity water spray. Reduction in the amount of total bacteria and 7 species representing early and late colonizers of various Socransky microbial complexes [10] was quantified by qPCR.

## Methods

### Planar optode imaging system

**Fig. 1:**
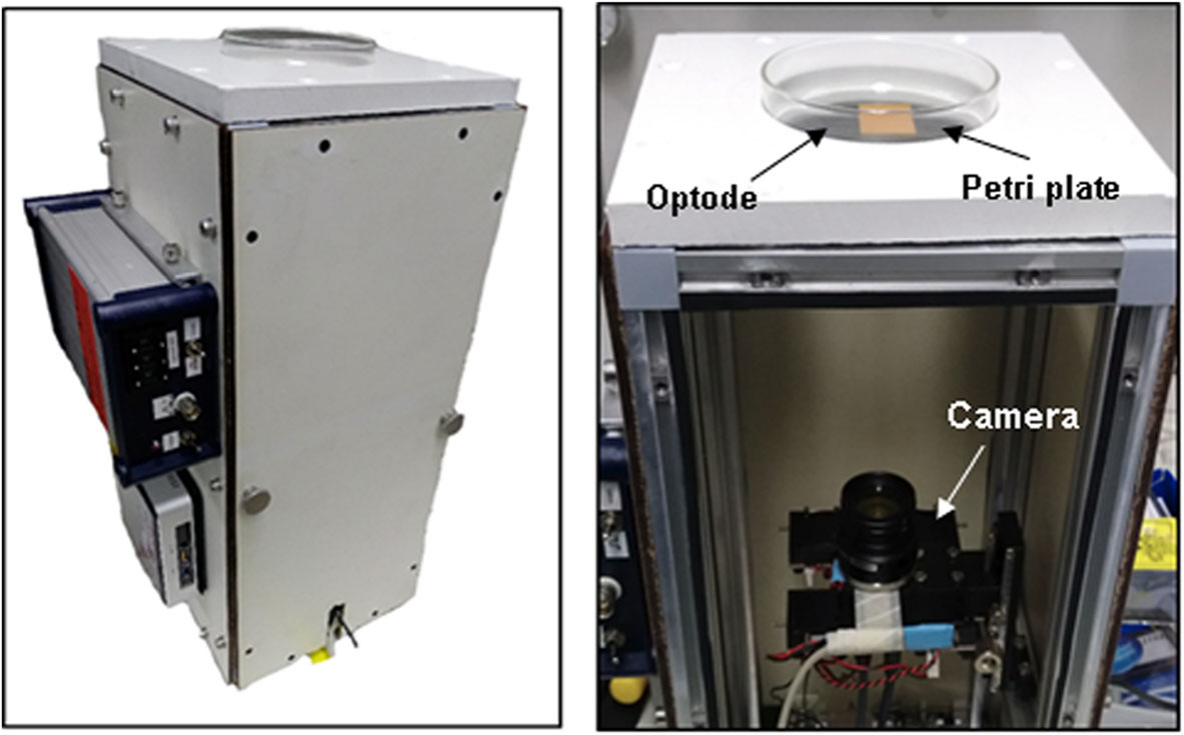
Optode imaging system. The imaging system consisted of a camera fitted with a 25 mm objective and mounted on a movable stage to adjust focus on to the opening at the top. The biofilm growth chambers were placed on the circular opening at the top and covered to eliminate ambient light during imaging. Strobe lights (455 nm LEDs) for fluorescence excitation were installed beside the imager. The left panel shows the system enclosed in a case to eliminate ambient light and the right panel shows the configuration of the system components and the sample

The planar optode imaging instrumentation was developed at the Max Planck Institute in Bremen, Germany. The setup comprised of a 45 cm × 20 cm × 20 cm illumination and imaging unit (Fig. 1). It housed a 8.8MP color camera (Flea3 USB from FLIR Systems Inc.), mounted on a linear stage to aid in focusing the field of view on to a sample opening at the top of housing. The camera was equipped with a 25 mm objective lens fitted with a 530 nm long-pass filter (Schott, North America). The top opening allowed samples to be imaged from below with controlled LED illumination (455 nm with a cut-off filter at 470 nm) which were positioned within the housing. Ambient light was eliminated by covering the opening with aluminum foil. The spatial resolution in the focal plane was 25 μm/pixel.

### Optode manufacture

The optode foils were prepared according to Larsen et al. [25]. However, a lipophilic coumarin dye Bu_3_Coum [26] was used instead of Macrolex yellow in order to avoid leaching of the antenna dye out of the polymer. The sensor material contained Bu_3_Coum (1.5% wt.), an oxygen indicator Pt (II) porphyrin (0.75% wt., Frontier Scientific, frontiersci.com) embedded in polystyrene (MW 250000) along with 50% by wt. of TiO_2_ nanoparticles (P170, Kemira, Finland). This mixture was dissolved in chloroform (4.5 g per 300 mg polystyrene) (Sigma, USA) and coated in a 2 μm thick layer on a clear polyethylene terephthalate sheet (Melinex 505, Pütz, Germany). The optodes were then stored dry and in the dark until used for the sample measurement setup.

### Calibration of the optodes

The ratio of the red and green channels in the color images of the optode can be calibrated for dissolved oxygen (DO) using a modified Stern-Volmer equation [25, 27]. The response is not linear but follows an exponential decay and thus a multi-point calibration is required to characterize the nature of the decay on a subset of the optodes. To achieve this, the signal of a de-aerated N_2_ sparged solution is measured simultaneously with a DO concentration from a microelectode system (Unisense) as the solution is sparged by bubbling with air and thus becomes re-oxygenated. Once the general response was characterized we used the Stern-Volmer equation to fit intermediate points between the 2 measured points; zero and 100% saturation on the other optodes. The 0% DO solution was made by adding 3 g of ascorbic acid (C_6_H_8_O_6_, 99.9%), (Sigma, USA) in 50 mL of tap water and adjusting pH to 11 with NaOH. A 100% DO solution was obtained by bubbling demineralized water with air for 10 min. Since the biofilms were to be grown in a modified brain heart infusion (M-BHI) broth, we used a conversion to account for the lower solubility of DO in BHI [28]. Calibration and measurements were performed at 22 °C Such optodes have been designed to be insensitive to pH, salinity and CO_2_ [27].

### Sterilization of optodes

After calibration, the optodes were sanitized in generic mouthwash containing 1.5% H_2_O_2_, 0.07% cetylpyridinium chloride for 30 min at room temperature. The optodes were then rinsed with dH_2_O, before being applied to the growth systems. We placed the optode in a petri dish with BHI (Sigma Aldrich, USA) and incubated it for 24 h at 37 °C to confirm sterility by inspection for turbidity and plating the media on BHI (Sigma Aldrich, USA) agar. If there was no turbidity and no colonies observed, we proceeded with the experiment.

### Collection of human saliva / plaque inoculum

This study was approved by the Ohio State University for Human Subject Research (Study Number: 2017H0016). Samples were collected from six consenting healthy adult donors, who did not self-report any known underlying chronic disease and were in good oral health using a protocol adapted from Nance et al. [29]. The donors had not received antibiotics for at least 3 months prior to collection. Collection of samples was performed in the morning for all volunteers. Volunteers were asked to refrain from eating after dinner the evening prior to and the morning of the collection. No oral hygiene was practiced for a minimum of 8 h prior to donating. To generate a saliva/plaque inoculum, 10 ml of stimulated saliva was collected in a sterile plastic 50 mL tube (Falcon, Thermo Fisher Scientific, Waltham, MA, USA). Plaque was recovered using a standard toothbrush from the teeth and tongue by brushing but using no toothpaste. The toothbrush was vortexed in 10 mL PBS for 3 min (Vortex-Genie® 2 mixer, Scientific Industries, Inc., Bohemia, N.Y., USA) to transfer the plaque from the brush to the PBS. Vortexing was conducted in an anaerobe chamber (Bactron, USA, with a 5% CO_2_, 5% H_2_ and 90% N_2_ headspace) to avoid overexposure of O_2_ sensitive anaerobes during the mixing. The bacteria was pelleted by centrifugation (10,000 g for 3 min) and resuspended in the pooled saliva. Saliva and plaque were kept on wet ice through the whole collection procedure. Glycerol was added to a final concentration of 25%. Aliquots of this suspension were stored in 1.5 mL cryogenic tubes (Thermo Fisher Scientific, USA) at − 80 °C.

### Growth media

A modified brain heart infusion (M-BHI) broth was used for cultivations. BHI broth (Sigma Aldrich, USA) was supplemented with 5 mg/L hemin (Alpha Aesar, USA), 1 mg/L menadione (MP Biomedicals, LLC, France), 0.1 g/L L-cysteine (Sigma, USA) and 1 g/L yeast extract (Sigma, USA). The growth media of current study was absent in. We were particularly interested to see if Gram negative strict anaerobes could survive and proliferate in a biofilm grown under an oxic headspace and we were concerned that adding sucrose would cause *S. mutans* and other acidogenic bacteruia to dominate since *P. gingivalis* is not acid tolerant [30].

### Biofilm growth systems

Three growth systems were used. First we used hydroxyapatite (HA) discs to determine the time sequence for the development of the bacterial community under our growth conditions. For the optode work, we used a modified dual chamber slide (Nunc Lab-Tek, USA) and a polystyrene Petri dish (Corning™ Falcon, Thermo Fisher Scientific), both fitted with optode foils.

### Incubation conditions

All biofilms were grown in a 5% CO_2_ incubator at 37 °C (Thermo Fisher Scientific). Conventionally, human plaque biofilms grown in vitro are done under anoxic conditions in an anaerobic chamber with as a low Eh (Redox potential) in the bulk fluid is required for the strict anaerobes [31]. However, we used an oxic headspace under the rationale that 1) the oral cavity is not anoxic and that the natural creation of anoxic microniches by the biofilm was more relevant and 2) to observe differences in DO at the base of the biofilm when disrupted by the microspray required an overlying oxic headspace, recapitulating the in vivo condition.

### Hydroxyapatite (HA) discs

**Fig. 2:**
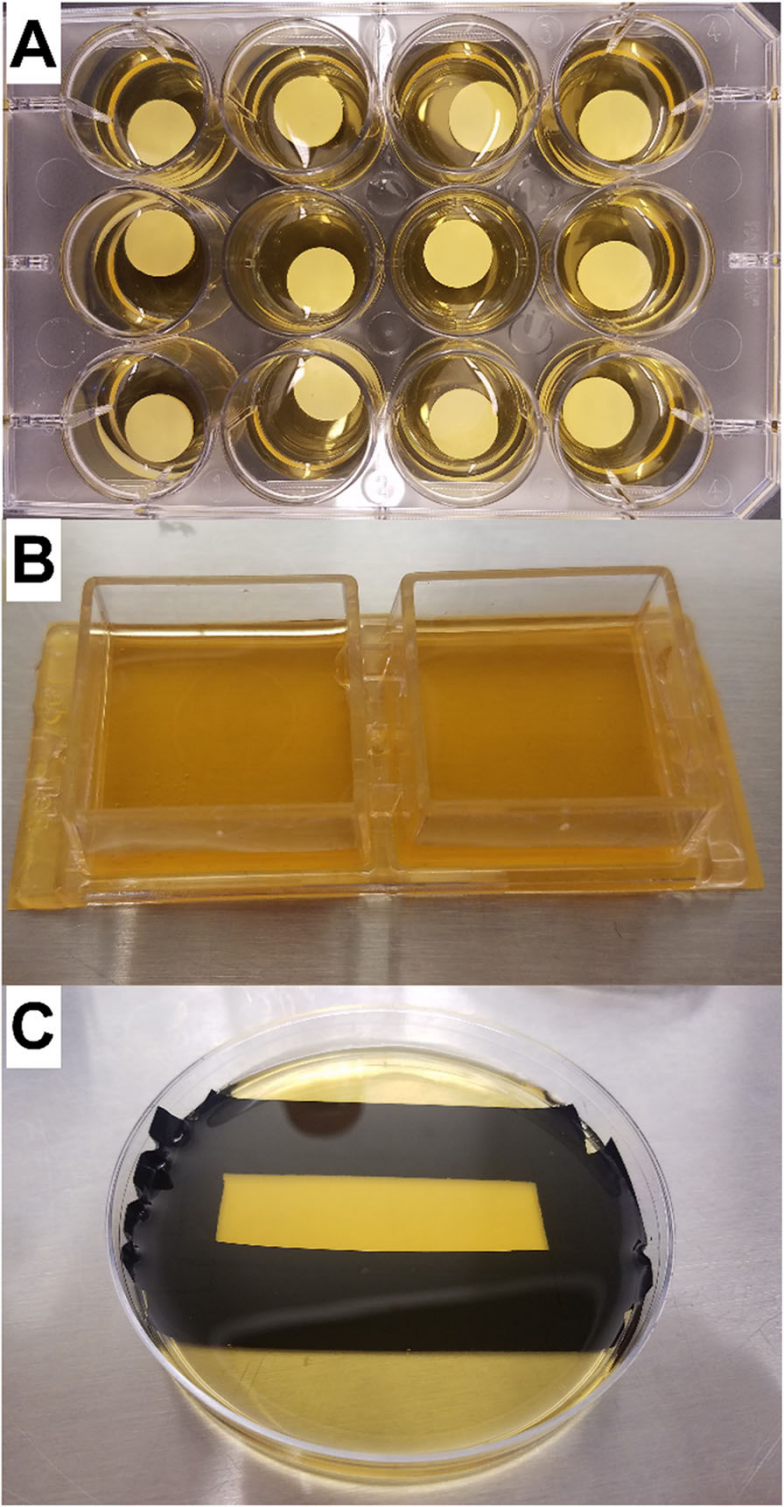
Growth systems for saliva/plaque biofilms. **a** HA discs placed in wells of a 12 well plate with 2 mL of medium. **b** Dual chamber growth system. To make the chamber the glass microscope slide was replaced with a planar optode (yellowish amber) which was glued to the chambers with silicone. Two mL of medium was held in each well. **c** Petri plate growth system. The optode was fixed to the bottom of the Petri plate using electrical tape to prevent the seepage of medium underneath the optode. The optode was submerged under 5 mL of growth medium

One cm diameter hydroxyapatite discs (BioSurface Technologies, Bozeman, MT) were placed into each well of a 12 well plate (Falcon, corning, USA) (Fig. 2a). Then 2 mL of sterile of M-BHI was added to each well followed by 500 μL of the saliva/plaque inoculum. The biofilm was grown for 4 days with daily media exchanges. At each day a triplicate set of dics were sacrificed to yield DNA for 16S RNA gene phylogenetic analysis (see below for details) by transferring them to 50 mL tubes (Falcon, Thermo Fisher Scientific, USA) with 5 mL of sterile phosphate-buffered saline (PBS, Gibco, Thermo Fisher Scientific). Biofilm was removed by sonicating in a sonicator bath (Model # 97043–964, VWR International, West Chester, PA, USA) for 3 min. The supernatant was then centrifuged (Legend micro 21, Thermo Fisher Scientific, USA) at 10,000 g for 10 min. The supernatant was discarded, and the pellet used for DNA extraction.

### Dual chamber system

In order to confine the biofilm to a defined area for the shooting experiment, a 2-well chamber slide with removable wells was used (Nunc™ Lab-Tek™ II Chamber Slide™ System, Thermo Fisher Scientific) (Fig. 2b). Each chamber was 2 × 2 cm with a volume of 2 mL. The cover slip that formed the base of the chamber was removed and an optode foil 22 mm × 44 mm was glued in its place using silicon sealer. One chamber was used as an “untreated” control and the other as the experimental HVM treated chamber. The microspray shooting was performed in a laminar flow cupboard to contain possible aerosols. However, the microspray was so forceful in the small chamber that it displaced almost all of the reservoir liquid making the system messy and difficult to work with. Therefore, we also used a larger Petri plate system. The DO measurements were limited due to resources and we were only able to use one run for the dual chamber and Petri plate systems each.

### Petri plate system

To overcome the problem of losing the water phase during HVM treatment, we used a 9 cm dia. Petri dish (Corning™ Falcon, Thermo Fisher Scientific) to grow biofilms. A piece of optode film 22 mm × 44 mm was fixed to the bottom of the plate with electrical tape. To prevent the seepage of medium underneath the optode and to prevent the formation of bubbles between the optode and the bottom of the plate, it was important to tightly seal all four sides of the optode (Fig. 2c). Electrical tape worked well because of its pliability. Another advantage of using this larger container was that the impinging spray was allowed to spread radically rather than splashing back out of the chamber system. In addition, the larger area of the optode meant that the area of biofilm dislodged by the treated area could be clearly delineated from the surrounding undisturbed areas, thus allowing oxygen mapping over both areas. A further advantage was that the optode was supported by the underlying plate and so the optode remained better positioned during HVM treatment.

### Biofilm growth in the dual chamber and petri plate optode systems

The dual chamber and the Petri plate biofilms were grown under similar conditions with daily media exchanges and a daily inoculation from the pooled stock for a duration of 7 days. For the dual chamber system, 2 mL of sterile medium was added to each chamber and 200 μL of the stock inoculum was added to each well. For the Petri plate system, 5 mL of media was added and 500 μL of inoculum. After days 1, 2, 3, 4, 6 and 7, the DO was mapped using the optode system by transferring the growth chamber (either dual chamber or Petri plate) to the optode imager and imaged immediately after exchanging the media, to assure the media to be fully oxic. On days 6 and 7, the biofilm was exposed “shot” with a commercial HVM device using a single pulse (AirFloss, Philips Oral Healthcare). The device was filled with tap water to generate a water microspray which lasted approximately 60 ms and dispensed approximately 130 μl [21]. The HVM device was convenient for these studies since it could generate high velocity fluid flow while minimizing the volume of generated liquid waste [32, 33]. In the dual chamber system, one of the chambers was HVM treated while the other chamber was left as an untreated control. The tip of the HVM device was held approximately 1 cm from the surface of the optode during the microspray. DO images were taken before and after the treatment. After the final optode measurement on day 7, the biofilm growing on the optode in the Petri plate was removed for DNA extraction for identification of species and genera by PCR. A circular area of approximately 1 cm diameter in the HVM shot area was sampled by scraping with a sterile loop (Fisherbrand, disposable loop, Thermo Fisher Scientific). A similar area in the adjacent but undisturbed area was sampled in a similar manner to serve as an untreated control.

### Biofilm community analysis

To ensure the bacterial community in the saliva/plaque inoculum included representative species (Table 1), we used conventional agarose gel PCR and assessed their relative abundance semi-quantitatively by gel densitometry. For biofilms grown on the HA discs and on the planar optodes in the HVM treated areas and the undisturbed biofilms, we used quantitative PCR (qPCR) to determine the presence and relative abundance of the target species.

**Table 1:** Oligonucleotide PCR primer sets used to identify target species and genera in the biofilm by densiometric gel electrophoresis and qPCR. For *S. mutans* the *gtfB* gene encoding glucosyltransferase was used. The other primers were for various regions of the 16S rRNA gene

Sequence of primer (5′–3′)	Target and abbreviation	Product size	Reference
F, GTTGACAGCCGATGAAGAAGATGAA R, TTCTCAGCAAAAGTACCGTCCTCG	*S. oralis* (So)	81 bp	[34]
F, GCCTACAGCTCAGAGATGCTATTCT R, GCCATACACCACTCATGAATTGA	*S. mutans* (Sm)	114 bp	[35]
F, GGTGTTGTTTGACCCGTTCAG R, AGTCCATCCCACGAGCACAG	*S. gordonii* (Sg)	96 bp	[35]
F,ATGTGGGTCTGACCTGCTGC^c^ R,CAAAGTCGATCACGCTCCG^c^	*A. viscosus* (Av)^c^	96 bp	[35]
F, GGATAGATGAAAGGTGGCCTCT^a^ R, CCAACTAGCTAATCAGACGCAAT^a^	*V. parvula* (Vp)^a^	72 bp	[36]
F, CCGTGATGGGATGGAAACTGC^b^ R, CCTTCGCCACTGGTGTTCTTC^b^	*Veillonella.* spp. (Vspp)^b^	102 bp	[37]
F, CGCAGAAGGTGAAAGTCCTGTAT R, TGGTCCTCACTGATTCACACAGA	*Fusobacterium.*spp. (Fspp)	101 bp	[35]
F, TAC CCATCGTCG CCTTGGT R, CGGACTAAAACCGCATACACTTG	*P. gingivalis* (Pg)	126 bp	[35]
F, CGCTAGTAATCGTGGATCAGAATG R, TGTGACGGGCGGTGTGTA	Eubacteria (Uni)	69 bp	[35]

^a^used in conventional PCR

^b^used in qPCR

^c^due to uncertainty of taxonomic identification of *A. viscosus* with respect to identification of this species in human strains we denote this species in quotation marks following Könönen et al. [38]

### DNA extraction

DNA from the saliva/plaque inoculum was extracted using a boiling method, which is a simple and cheap method that has been shown to be effective for human dental plaque, although for quantification is less sensitive than qPCR. In this method the scraped biofilm was boiled in double-distilled water (ddH2O) for 10 min. and then chilled for 2 min at 20 °C. The sample was then centrifuged (Legend micro 21, Thermo Fischer Scientific, USA) at 16,000 g for 10 min at room temperature. Purity of the extracted DNA was based on the 260/280 nm optical density (OD) ratio by spectrophotometry (NanoDrop 1000, Thermo Fisher Scientific). For qPCR, DNA was extracted immediately after the shot. For qPCR, we used the established method used in the “Human Oral Microbiome Identification using Next Generation Sequencing” (HOMINGS) protocols developed at the Forsyth Institute, for which we used an Epicenter, Puremaster Kit (Epicenter, Madison, WI) to extract DNA from biofilm [39].

### Conventional PCR and densitometry for the saliva/plaque inoculum

PCR conditions (annealing temperature and numbers of PCR cycles) were optimized to identify 5 of the 7 target species and genera using the primer sets shown in Table 1. Amplification was performed in a 25 μl mixture containing Mg^2+^, dNTPs, and recombinant Taq DNA Polymerase for routine PCR of fragments up to 5 kb (Invitrogen, USA), 10 μM forward and reverse primers and 2 μl bacterial DNA extract in a thermal cycler (MyCycler™ Thermal Cycler system, BioRad). PCR was carried out using the following conditions: an initial denaturation step for 4 min at 94 °C, with 45 cycles of 30 s at 95 °C, 1 min at 58 °C and 30 s at 72 °C, followed by 5 min at 72 °C. Agarose gel (Sigma, USA) was prepared at a concentration of 1.5% (w/v) in 60 ml Tris-Borate Buffer (TBE). One μl of 10 μg/ml ethidium bromide (Sigma, USA) was incorporated into the gel to make a final concentration of 0.5 μg/ml and electrophoresed at 90 V for 60 min. The DNA bands were visualized using a gel documentation system (ChemiDoc XRS, Bio-Rad, USA) under ultraviolet (UV) illumination.

### qPCR for analysis of HA and optode grown biofilms

qPCR was performed using an iQ5 real time PCR detection system (CFX96, Bio-Rad, USA). Each PCR was performed in a total volume of 20 μL containing 2 μl of 10× iQ SYBR® Green Supermix (Bio-Rad, Hercules, CA), 0.5 μmol/L each of forward and reverse primers, 7 μl of ddH2O water, and 1 μl (10 ng) of template DNA., depending on the ratio of the individual bacterial species within the biofilm. We used SYBR green rather than a TaqMan probe to make the assay more economical after Yamashita and Carrouel et al. [40, 41], however, we note limitations to this method, it is less specific and sensitive. Therefore, when using this method it is necessary to carefully check the primer concentration, buffer composition and denaturation and annealing times to verify a robust qPCR reaction. The qPCR was carried out with an initial incubation of 2 min at 95 °C, followed by 45 cycles of denaturation for 15 s at 95 °C, annealing for 1 min at 60 °C, melting curve 65 °C to 95 °C incremented by 0.5 °C every 0.05 s. Standard curves were generated for all the target bacteria using DNA from pure cultures and species specific DNA primers. Ten-fold serial dilutions of genomic DNA (DNA from 10^7^ to 10^1^ cells), from pure bacterial cultures provided seven data points for standard curve generation. In each standard curve, the concentration of standard sample (known amount of DNA/DNA from known number of bacteria) was plotted against its crossing points. Quantification of the individual target bacteria and total bacteria from the experimental samples were calculated using the standard curves. For each species, a standard curve was generated using defined concentrations of genomic input DNA (10-fold serial dilution). The genomic DNA amount of the target species or genera in the unknown sample was calculated from the standard curve. For the HA grown biofilms triplicate discs (biological replicates) were sacrificed for qPCR analysis on each of the 4 days. For the optode experiments biofilms were grown for 6 days with daily nutrient exchanges and then shot with the HVM on days 6 and 7. Triplicate unshot optodes were used for comparative controls. Three biological replicates containing three technical replicates were used in this set up experiment. Data were analyzed using Bio-Rad CFX manager software 3.1. qPCR analysis resulted in Ct-values which were converted to copy number using a calibration curve and graphics were made using GraphPad Prism 8.0 (GraphPad Software, Inc., San Diego, CA). The student t-test was used for qPCR data analysis. *P* < 0.05 was considered significantly different.

### Confocal laser scanning microscope (CLSM)

CLSM was used to measure the thickness and visualize the structure of the biofilm on the HA discs and the optodes in the undisturbed areas and the HVM “shot” areas. The dental/plaque biofilm was grown for 7 days on both surfaces, and shot on day 6 and 7. Briefly, on day 7 biofilms on the HA discs and optodes were rinsed two times with sterile saline to remove loose bacteria, and then “shot” with a HMV. Immediately after the shot, the samples rinsed with sterile water and the remaining bacteria were stained with 2.5 μM SYTO 9 green fluorescent nucleic acid stain (480/500 nm; ThermoFisher Scientific) with a 30 min incubation in the dark at room temperature [42]. After incubation imaging was performed using an Olympus FV1000-MPE upright confocal/multiphoton microscope (Olympus Corp., USA) equipped with a × 25, 1.05-numerical-aperture water immersion dipping objective lens. The excitation wavelength was 488 nm, and the emission wavelength filter for SYTO 9 was a 500/530 OlyMPFC1 filter. Each biofilm was scanned at three randomly selected positions in the HVM and undisturbed areas on three biological replicates of HA discs and one optode. The XY-*Z* series were generated by optical sectioning at each of these positions. Graphics were made using GraphPad Prism 8.0 (GraphPad Software, Inc., San Diego, CA). COMSTAT2 [43] was used to measure the maximum thickness of the biofilm and student t-test run for data analysis. *P* < 0.05 was considered significantly different.

## Results

### Presence of the target species in the inoculum

The pooled saliva/plaque inoculum was positive for all the targeted species and genera representing both aerobic and anaerobic species (Supplemental Fig. 1).

### Biofilms grown on the HA discs

**Fig. 3:**
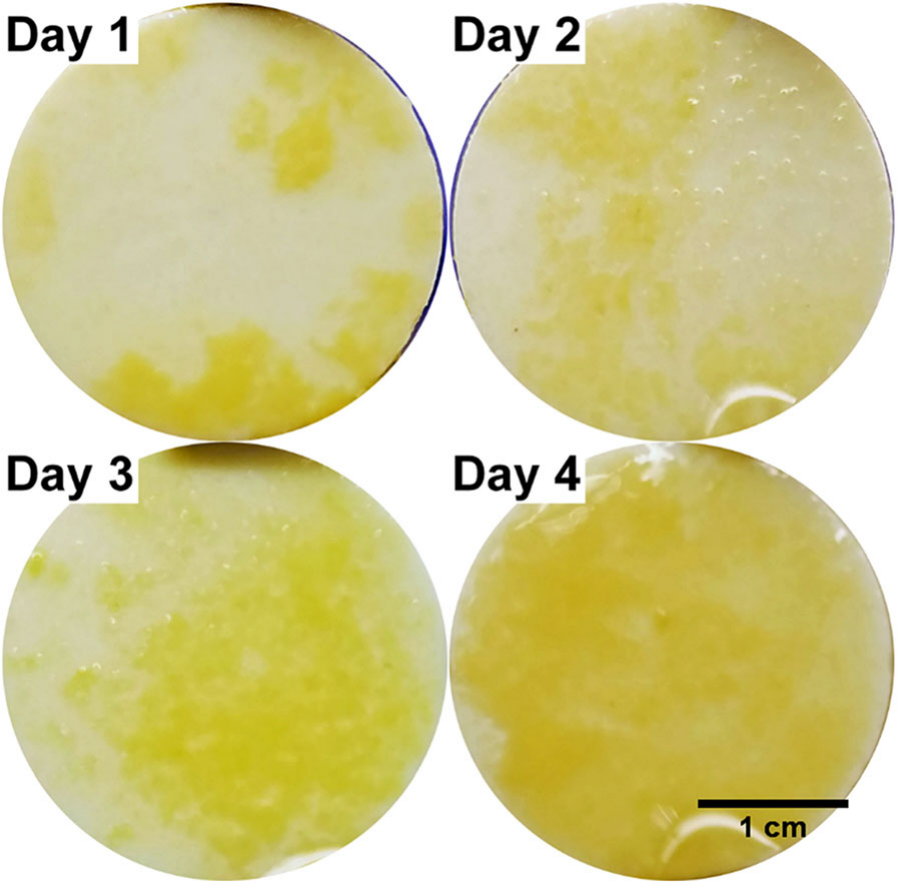
Progression of biofilm development on HA discs. Over the 4 days of growth the biofilm progressed in surface coverage on the disc, becoming progressively more uniform by 4 days. Contrast enhanced (Adobe Photoshop Elements 15) to more clearly show the biofilm against the background. The dark region near the top of the disc and the arc near the bottom are artefacts from the forceps holding the discs

**Fig. 4:**
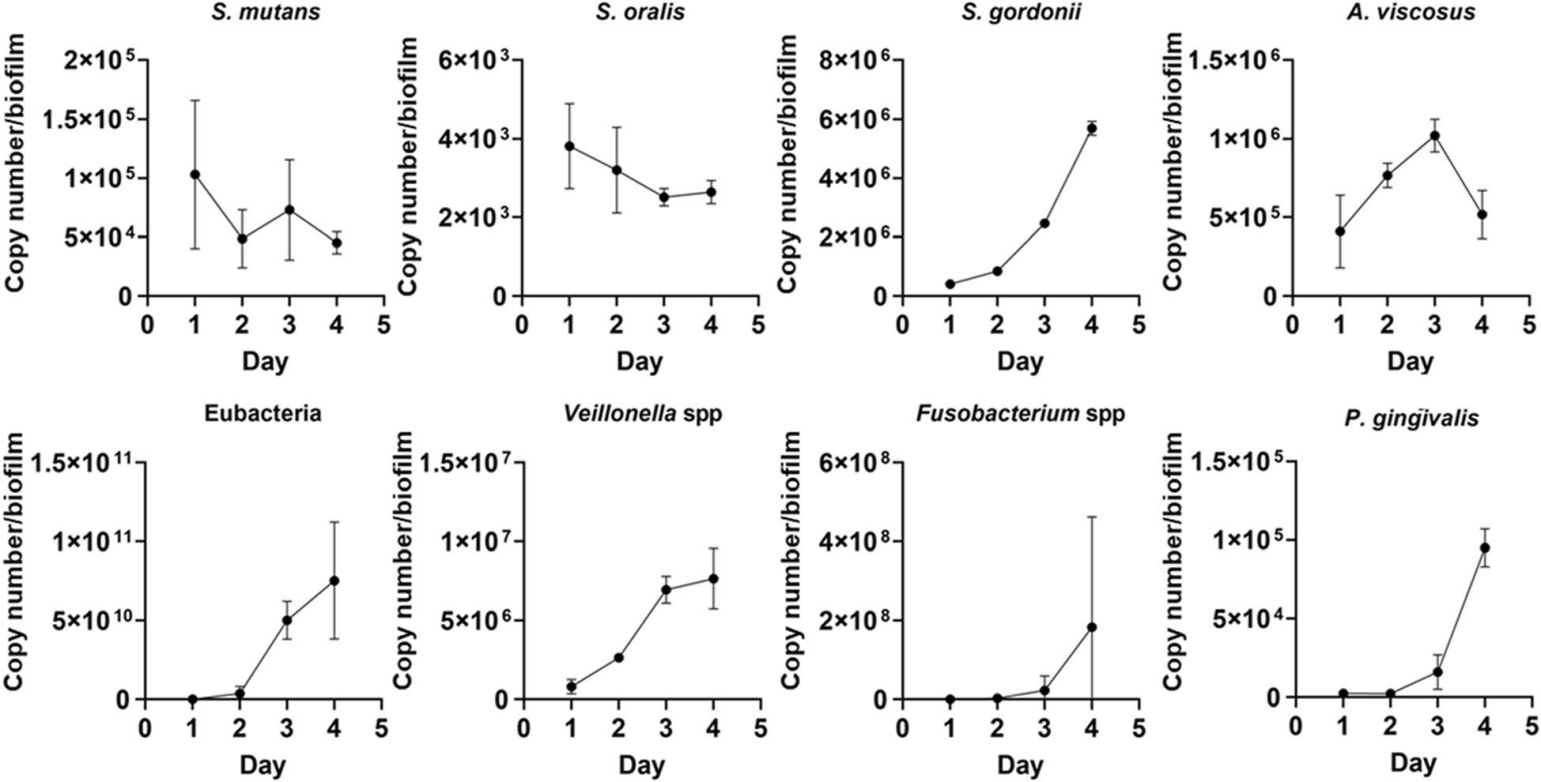
Development of the biofilm community on the HA discs by qPCR. In the day 1 biofilm *Veillonella* spp. *Fusobacteria* spp. and *S. oralis* were the most dominant of the target species. There was a general trending increase in all of the anaerobic species between days 1 and 3. Data were mean and 1SD

Macroscopically the biofilm grown on the optodes and the HA were heterogeneous and consisted of distinct microcolonies of up to 2 mm in diameter surrounded by a more uniform cream colored base film (Fig. 3). The biofilms were approximately 1 mm thick after 4 days. Over the course of 4 days of growth, there was a general increase in the biomass as demonstrated by the increase in copy number; however, individual species showed different trends (Fig. 4). After 1 day of growth, *Veillonella* spp. *Fusobacteria* spp. and *S. oralis* were the most dominant of the target species in the biofilm. There was small but steady decrease of the two streptococci over the 4 days while there was a general increase in the anaerobic species between days 1 and 3. At day 4, “*A. viscosus”*, *Veillonella* spp., *Fusobacterium* spp. and *P. gingivalis* showed a subsequent decrease of approximately 10 to 20%. In contrast there was an approximate 4 log increase in *Fusobacteria* spp. from days 3 to 4.

### Biofilms grown on the optodes

**Fig. 5:**
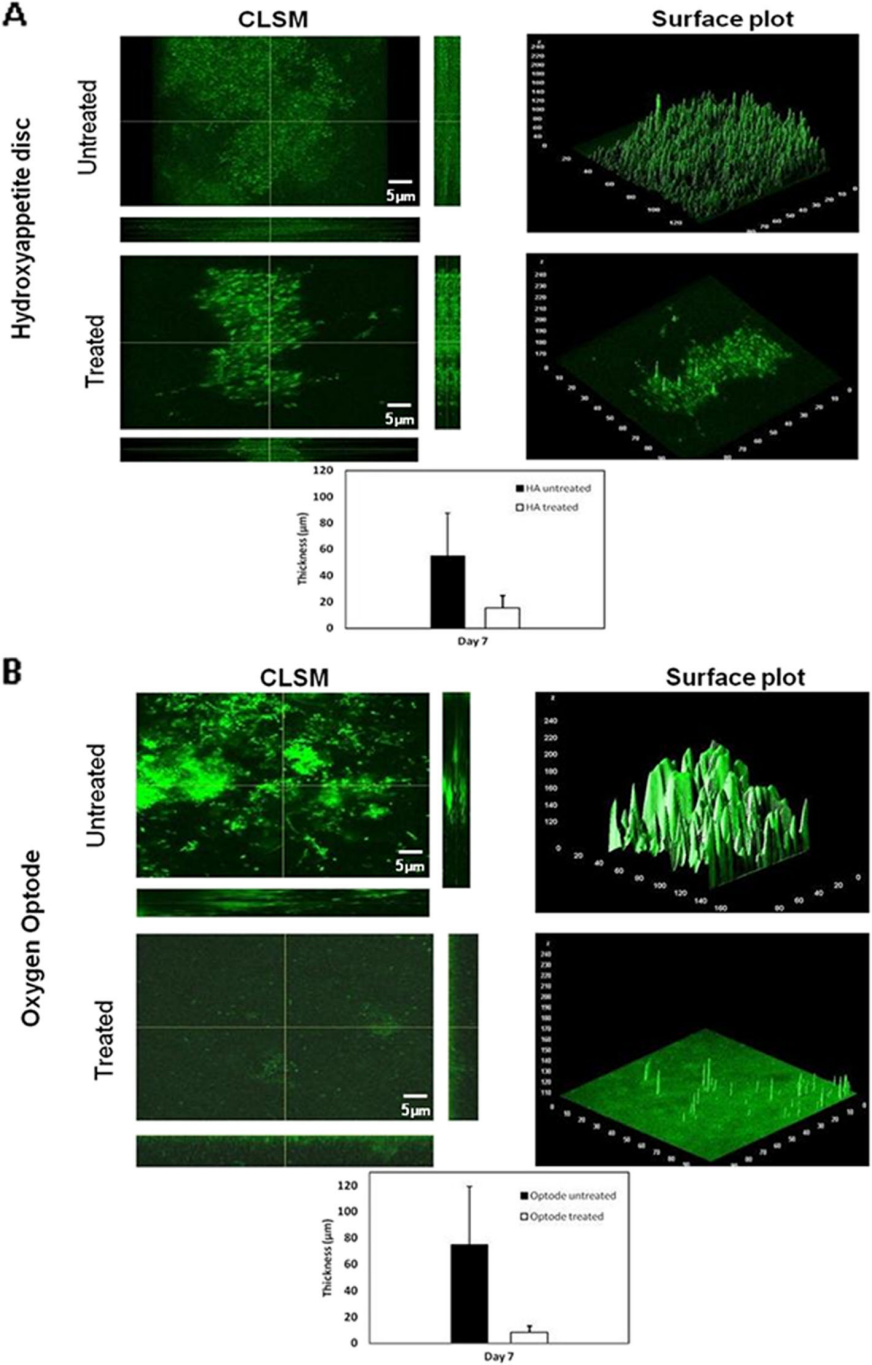
COMSTAT2 analysis of 7 day old saliva/plaque biofilm grown on the HA discs and an oxygen optode in the Petri plate system. Maximum biofilm thickness quantified with COMSTAT2 on HA discs (3 biological replicates each with 3 fields of view in each arm) and (**b**) and the oxygen optode (one optode with three fields of view in the HMV shot and undistrurbed biofilm areas). Differences in thickness between the HMV shot and undisturbed areas were statistically significant (*P* < 0.05 by unpaired Student’s t-test)

**Fig. 6:**
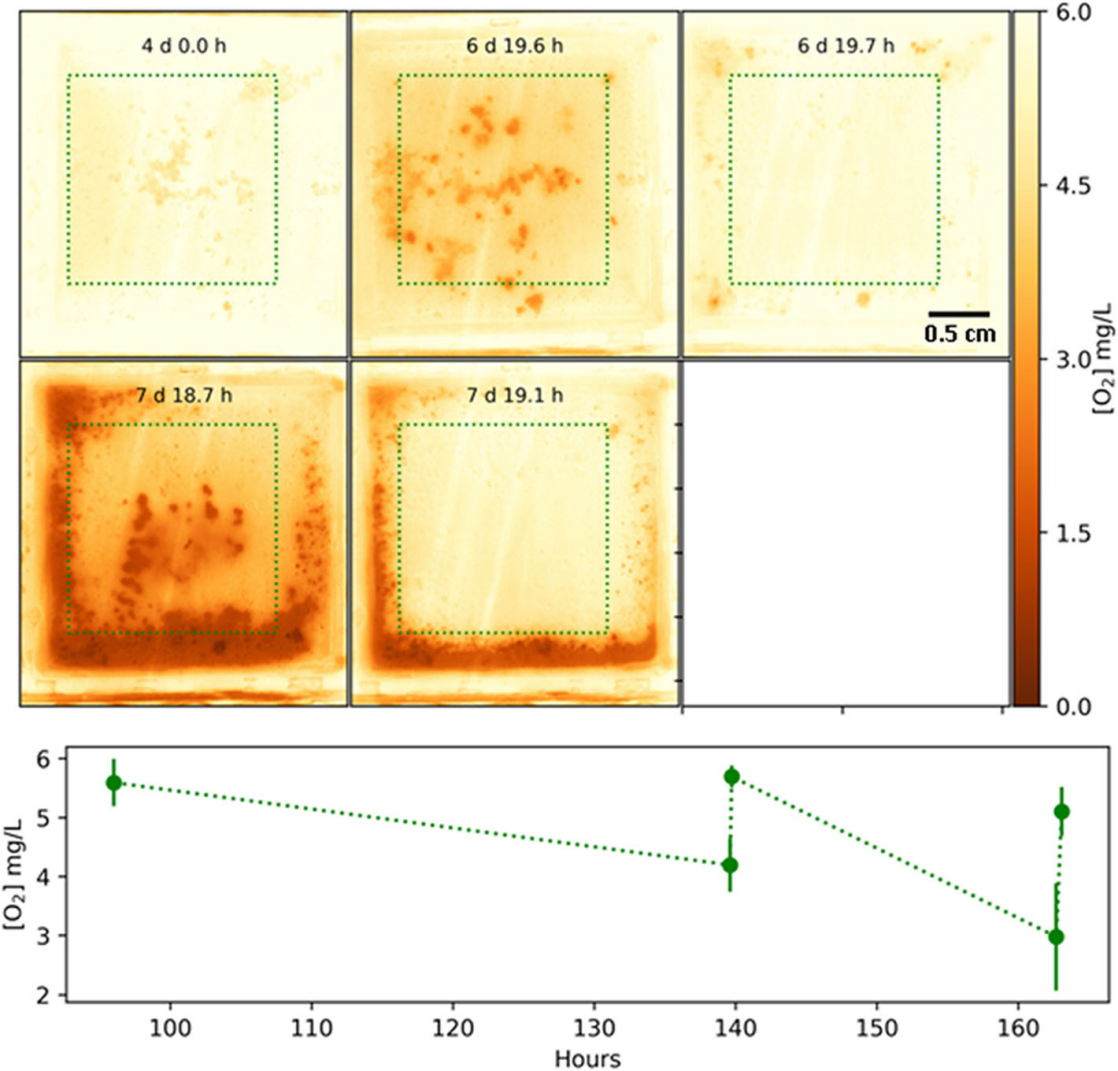
Dissolved oxygen in the dual chamber before and after HVM treatment. Top panels are a time series showing the DO distribution represented as a false-color image. The graph below shows the DO concentration across the shot area (marked with profile lines in the images) at the various time points. The biofilm was exposed to HVM treatment at 6 days and again at 7 days. The diameter of the area cleared by the HVM was approximately 1 cm

**Fig. 7:**
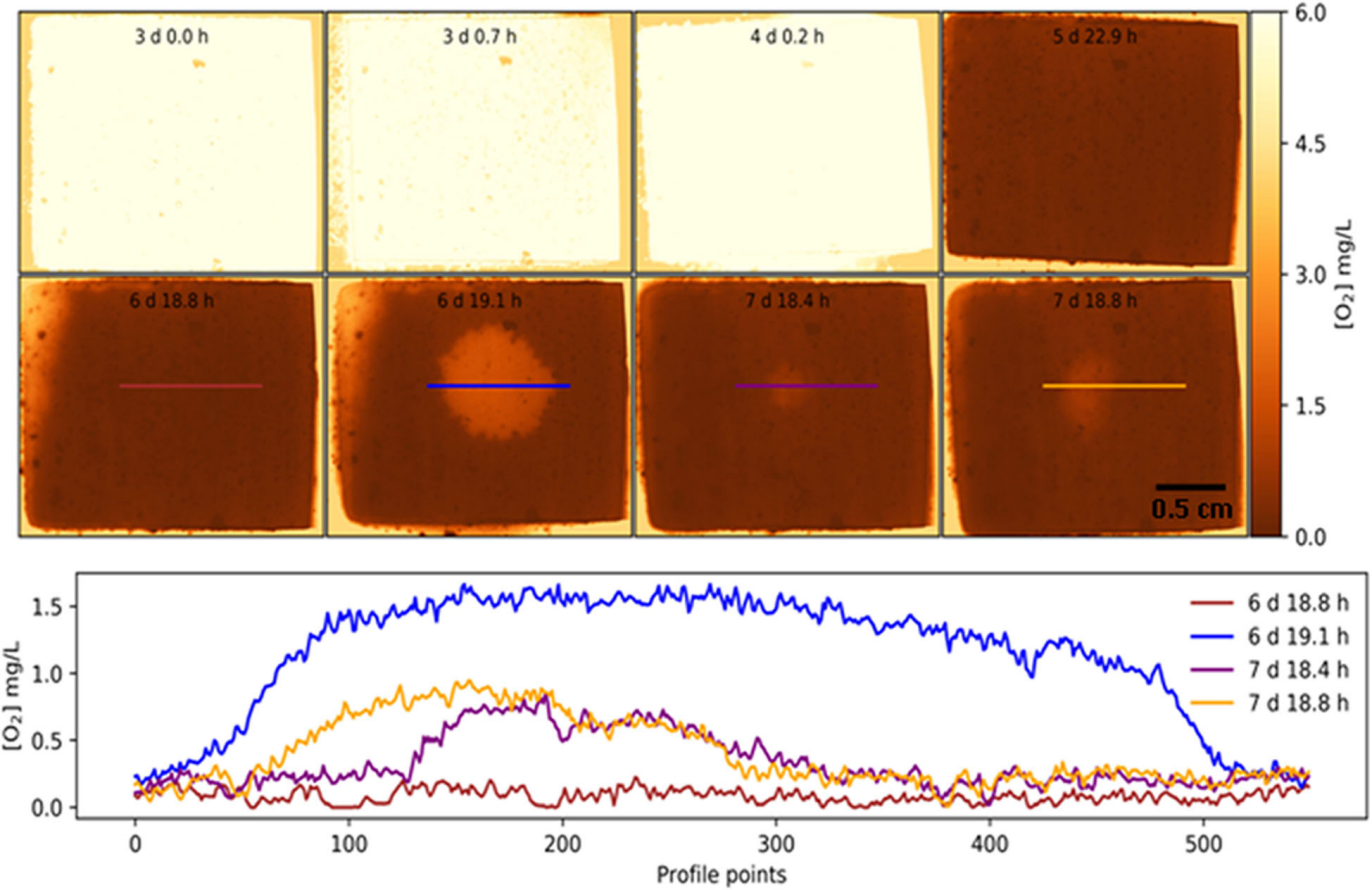
Dissolved oxygen profile across the HVM treated area in the Petri plate biofilm**.** Top panels are a time series showing the oxygen distribution represented as a color heat map. The graph below shows the DO concentration across the HVM cleared area at the various time points. The biofilm was exposed to HVM treatment at 6 days and again at 7 days. The diameter of the HVM cleared area was approximately 1 cm

The biofilm grown on the optode foils were similar in appearance to those grown on the HA discs and consisted of discrete microcolonies surrounded by a base film (Figs. 3 and 5). The larger microcolonies were approximately 1 to 2 mm diameter. After the biofilm was exposed to the HVM in the dual chamber system there was visible clearance of biofilm from the base of the chamber (Fig. 5). However, the HVM appeared to have forced biofilm to the chamber wall edges where it had accumulated (Fig. 6). In contrast the HVM treatment to the biofilm grown on the planar optode in the Petri plate created a well-defined circle of clearance, approximately 1 cm diameter, Epifluorescence microscopy showed that the larger aggregates had been removed from this area (Fig. 7).

### Thickness of biofilm

Confocal microscopy imaging and the surface plot in the HVM treated areas showed that biofilms on both the HA and optode surfaces were highly heterogeneous (Fig. 5). Such heterogeneity might explain different oxygen concentrations at the base of the biofilm. The average maximum (in a field of view) thickness of biofilms on the HA discs was 55 μm with a maximum thickness of 110 μm. Exposure to the HVM reduced the average maximum thickness to 15 μm with the maximum measured value of 31 μm. For the optodes the average maximum in the undisturbed region was 75 μm with a maximum thickness of 150 μm. Exposure to the HVM reduced the average maximum thickness to 8 μm with the maximum measured value of 16 μm. These reductions were statistically significant (*P* < 0.05).

### Dual chamber system

In the dual chamber the optode substratum was almost fully saturated with DO at 6.3 mg/L at the time of inoculation but dropped to 4.7 mg/L after 2 days growth (Fig. 6). However, after 2 days of growth anoxic regions of 1.5 mg/L were seen developing underneath the larger microcolonies. After HMV treatment to the right chamber the larger colonies were removed and the DO returned immediately to 6 mg/L. However, less oxic regions persisted around the edge of the chamber presumably caused by physiological activity of biofilm which had been removed from the central area of the chamber to the edges. After a further day of incubation there was notable accumulation of biofilm regrowth and microcolonies reappeared in the center and around the edge of the right chamber. The DO was reduced to around 1.0 mg/L. The second HVM treatment to the right chamber again removed the distinct biofilm microcolonies from the central region and the DO increased to 6 mg/L.

### Petri plate system

Similar to the dual chamber system, the optode substratum was close to 100% DO saturation at the time of inoculation. There was little change after 1 day but after 2 days the DO at the base of the biofilm had dropped from approximately 6 mg/L to 0.1 mg/L (Fig. 7). Unlike the dual chamber system, the DO distribution was fairly homogeneous since there were less large distinct microcolonies. After HVM treatment to the optode on day 6 the DO increased to 1.5 mg/L in the cleared circular region of 0.57 cm^2^ (0.85 cm diameter) where biofilm was removed. After a further days of incubation biofilm had grown back into the cleared region reducing it to 0.08 cm^2^ (0.32 cm diameter). The DO in this region had reduced to 1 mg/L but was still higher than prior to treatment (o.1 mg/L). After the second HVM treatment the cleared zone increased to 0.40 cm^2^ (0.72 cm diameter) which was less than had been achieved with the original HVM and the DO only increased to 1 mg/L the second time.

### Bacterial community analysis from the optode in the petri plate system

**Fig. 8:**
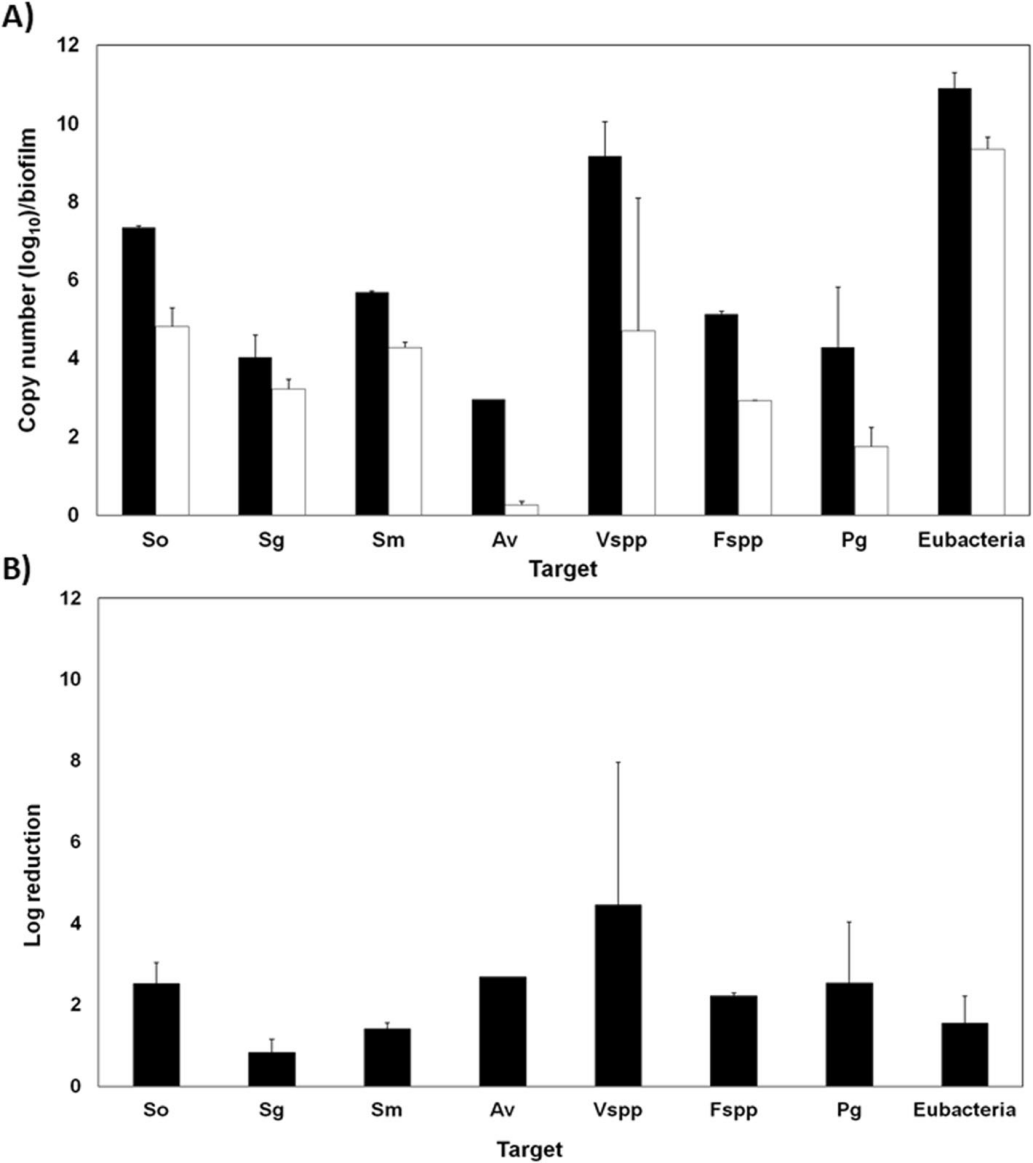
qPCR relative abundance of target bacterial species grown on the oxygen optodes exposed to the HVM shot area and an equivalent area in an unshot control**. a** Log _(10)_ copy number per biofilm of undisturbed and HVM treated regions of the biofilm. There was a significant reduction in the target species in the treated areas (*P* ≤ 0.05). Black: Untreated control biofilm, White: HMV treated biofilm. **b** Log relative reduction in species abundance after HVM treatment. Data were log _10_ transformed and presented as geometric mean and 1 SD

qPCR showed clear differences in the amount of DNA amplified from the HVM treated area compared to the undisturbed (untreated control) area (Fig. 8a). In the untreated area the total copy number was 2.16 × 10^11^. After HVM treatment the total cell copy number was reduced to 2.62 × 10^09^, an approximate 2 log reduction (Fig. 8b). All of the targeted species showed a reduction ranging from an approximate 1 log reduction for *S. gordonii* to over a 3 log reduction for *Veillonella* spp. There was no clear trend indicating whether the anaerobic species were selectively removed relative to the facultative anaerobic Streptococci.

## Discussion

Here we report a method of using planar optodes to measure the influence of mechanical disruption of dental biofilms by HVM on DO concentration at the base of the biofilm. Previously, we have measured DO profiles in ex-situ human dental biofilms with microelectrodes to show that anoxic regions are created near the base of the biofilm and although antimicrobial dentifrices reduce microbial activity in the upper parts of the biofilm the anoxic regions remained active [8]. While DO microelectrodes are extremely useful for measuring respiration rates and the development of anoxic conditions under varying conditions at different depths in biofilms, they do not easily provide a 2D oxygen distribution map of DO concentrations at the base of the biofilm. They are also very fragile and cannot be used during the application of mechanical disruption technologies due to issues with vibration and breakage. Ratiometric dyes have been used to show anoxia at the base of undisturbed dental biofilms [44] but mechanical disruption will wash such dyes out complicating interpretation of the results. Planar optodes have been previously utilized to map oxygen in sediments [45] and heterotrophic biofilms as a function of flow rate [46] but not to our knowledge in dental biofilms undergoing mechanical disruption.

Biofilm developed on the DO optodes had a similar appearance to that on the HA and polystyrene surfaces. PCR showed that the inoculum contained both early colonizers as well as anaerobic periodontal pathogens. The relative increase in anaerobic species over the 4 days of growth on the HA discs demonstrated that these organisms were still viable even though special considerations with respect to maintaining an anoxic environment were not taken during collection or culturing. It is possible that biofilm aggregates in the saliva or detached during brushing were already providing an anoxic niche, which allowed pathogen numbers to increase as the biofilm developed. Obligate anaerobic species have previously been reported in biofilms grown in flow cells with no special provision to impose an anoxic environment on the culture [29] suggesting that these organisms can occupy that anoxic niches which develop naturally through oxygen consumption from facultative species in the biofilm, allowing anaerobes to thrive in vitro as they might in vivo. In the first few days of biofilm formation of the monitored species *Veillonella .*spp. dominated in the biofilm. In human plaque the streptococci are known to dominate the supragingival plaque [47, 48] however, we only monitored two representative streptococci species and so we likely underestimated the relative proportions. The decrease in streptococci and the increase in *Fusobacterium* spp. is consistent with the succession seen in natural oral populations.

Treating with the HVM device showed significant mechanical disruption of the biofilm in both optode growth systems and larger microcolonies were visibly removed in the treated areas. After treatment the DO concentration was immediately elevated by 2 fold in the dual chamber system and 10 fold in the Petri plate system rendering the biofilm base from anoxic to oxic. A reason for the greater difference in the Petri plate system might be that the smaller dimensions of walls of the dual chamber system retained detached biofilm within the cleared zone, and particularly around the edges, where it would still have been consuming oxygen in the immediate vicinity, influencing the oxygen concentration. The relative abundance of bacteria in the HVM treated and untreated areas of the same biofilm showed the most pronounced changes in *Fusobaterium* spp. and *P. gingivalis,* which showed a 6-fold reduction and complete removal, respectively. *P. gingivalis* and *F. nucleatum* belong to the group of strictly anaerobic bacteria associated with periodontal diseases [49]. Due to its numerous putative virulence factors and highly inflammatory nature, *P. gingivalis* is considered one of the major periodontopathic bacteria [50] and *F. nucleatum* is important due to its extensive co-aggregating capacity which bridges commensal and periodontal species in the biofilm [51].

Ideally, mechanical disruption by any interproximal plaque management device would remove all biofilm so that between dental cleanings, the succession is arrested to the early colonizers, however complete removal by mechanical methods is not achievable by any home use device. Recently, we discovered in an *S. mutans* interproximal model that HVM not only removed significant amounts of biofilm from but also caused the remaining biofilm to liquefy allowing more efficient delivery of dentifrices [52]. It is likely that repeated disruption of the biofilm will have an additional therapeutic effect of disrupting anoxic and acidic regions thus shifting the microbes from an anaerobic pathogenic community to a more benign commensal community, thus explaining the observed therapeutic effect [53]. By growing mixed species biofilms, containing obligate anaerobes under an aerobic headspace allowed us to measure how mechanical disruption influences oxygen concentration at the base of the biofilm. However, we point out caution in extrapolating our findings to clinical efficacy. The biofilm plaque model used in this study is was grown in nutrient-rich medium in the absence of real world host factors. Also the device is intended for interproximal delivery. Although jet impingement will create radial shear stresses as the spray radiates outwards from the site of impact inevitably there will be differences between the shear forces generated in our in vitro systems and those generated in the IP spaces of the human mouth. Therefore, it is important in future work to determine how the impact of repeated exposure to the microsprays influences microbial communities in longer term studies and how these data compare with those found in human clinical studies.

## Conclusions

In conclusion, we have shown that high velocity microsprays removed a sufficient amount of biofilm to disrupt the anoxic region at the biofilm-surface interface. The management of the biofilm microenvironment through repeated HVM treatment has the potential to select against the highly virulent and immunogenic anaerobic periopathogens.

## Supplementary information

**Additional file 1: Figure S1.** Presence of representative species and genera in saliva/plaque inoculum. Gel electrophoresis of 16S amplicons from the in vitro biofilms showing presence of the target species and genera. Lane 2–3: two technical replicates of sample, N: Negative control (without DNA), Lane 1: Positive controls with DNA extracted from pure cultures of *P. gingivalis* 33277, *S. oralis* 10557, *F. nucleatum* 10953, *A. viscosus* 43146, *V. parvula* 17745, *S. mutans* UA159 and *S. gordonii* DL1. due to uncertainty of taxonomic identification of *A. viscosus* with respect to identification of this species in human strains we denote this species in quotation marks following Könönen et al. 2015 [38].

## Data Availability

All data generated or analyzed during this study are included in this published article and its supplementary information files. Access to raw data can be acquired by connecting to the corresponding author via email.

## Abbreviations

DO: Dissolved oxygen (DO)
HVM: High velocity microspray
M-BHI: Modified brain heart infusion
qPCR: Quantitative PCR
OD: Optical density
HOMINGS: Human oral microbiome identification using next generation sequencing
TBE: Tris-borate Buffer
UV: Ultraviolet
HA: Hydroxyapatite (HA)
CLSM: Confocal laser scanning microscope (CLSM)
SO: *S. oralis*
Sm: *S. mutans*
Sg: *S. gordonii*
Av: *A. viscosus*
Vspp: *Veillonella* spp.
Vp: *V. parvula*
Fspp: *Fusobacterium* spp.
Pg: *P. gingivalis*
Uni: Universal
