# Correction to: Use of an oxygen planar optode to assess the effect of high velocity microsprays on oxygen penetration in a human dental biofilms in-vitro

**DOI:** 10.1186/s12903-020-01236-x

**Published:** 2020-09-04

**Authors:** Yalda Khosravi, Rala D. P. Kandukuri, Sara R. Palmer, Erin S. Gloag, Sergey M. Borisov, E. Michelle Starke, Marilyn T. Ward, Purnima Kumar, Dirk de Beer, Arjun Chennu, Paul Stoodley

**Affiliations:** 1 Department of Microbial Infection and Immunity, Ohio State University, Columbus, USA; 2 Max Planck Institute for Marine Microbiology, Bremen, Germany; 3 College of Dentistry, The Ohio State University, Columbus, OH USA; 4 Institute of Analytical Chemistry and Food Chemistry Graz University of Technology Stremayrgasse, Graz, Austria; 5 Philips Oral Healthcare, Bothell, Washington 98021 USA; 6 Department Orthopaedics, Ohio State University, Columbus, USA; 7 National Centre for Advanced Tribology (nCATS), Mechanical Engineering, University of Southampton, Southampton, UK

**Correction to: BMC Oral Health 20, 230 (2020)**

**https://doi.org/10.1186/s12903-020-01217-0**

After publication of the original article [1], the authors identified errors in the authors’ names of Sara R. Palmer and Rala D.P. Kandukuri.

The incorrect authors’ names are: Sara Palmer and Raja Durga Prasad Kandukuri.

The correct authors’ names are: Sara R. Palmer and Rala D.P. Kandukuri

The author group has been updated above and the original article [1] has been corrected.
