# Supplementary figures and images for: Use of an oxygen planar optode to assess the effect of high velocity microsprays on oxygen penetration in a human dental biofilms in-vitro
Source: BMC Oral Health. 2020 Aug 21;20:230. doi: 10.1186/s12903-020-01217-0 (PMC7441732; doi:10.1186/s12903-020-01217-0)

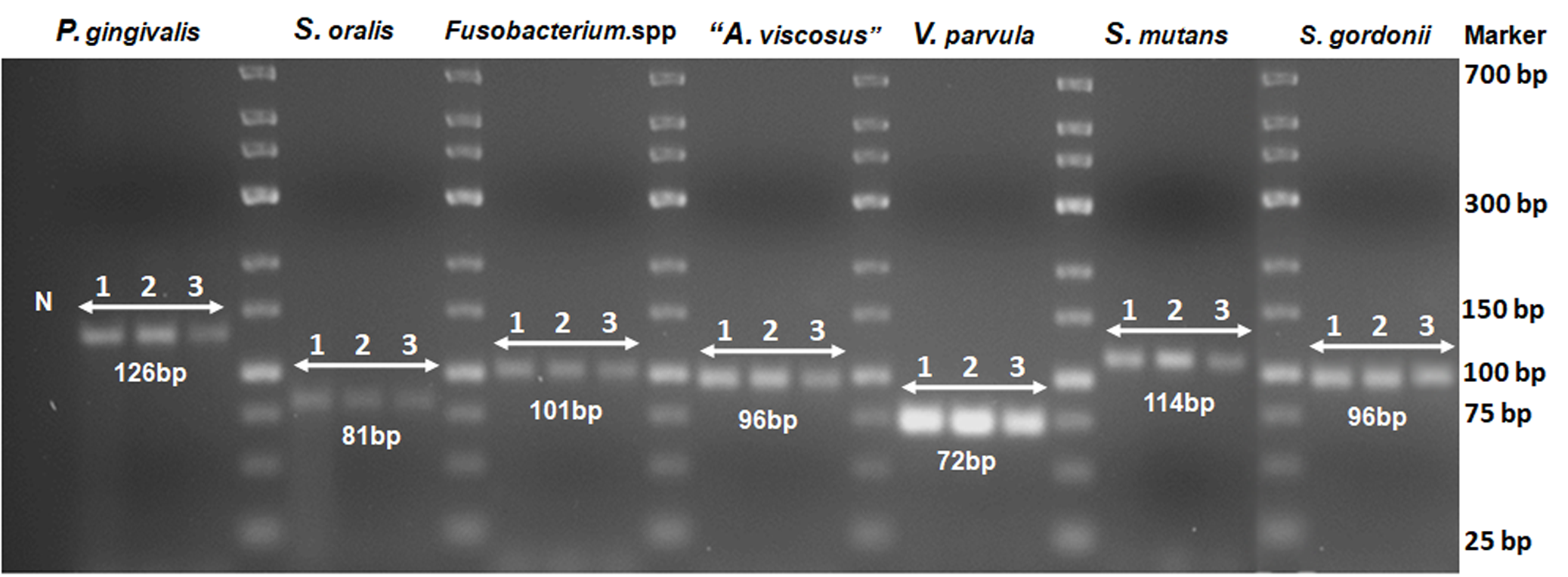

Supplement: Supplementary file 1 — Additional file 1: Figure S1. Presence of representative species and genera in saliva/plaque inoculum. Gel electrophoresis of 16S amplicons from the in vitro biofilms showing presence of the target species and genera. Lane 2–3: two technical replicates of sample, N: Negative control (without DNA), Lane 1: Positive controls with DNA extracted from pure cultures of P. gingivalis 33277, S. oralis 10557, F. nucleatum 10953, A. viscosus 43146, V. parvula 17745, S. mutans UA159 and S. gordonii DL1. due to uncertainty of taxonomic identification of A. viscosus with respect to identification of this species in human strains we denote this species in quotation marks following Könönen et al. 2015 [38]. [file 12903_2020_1217_MOESM1_ESM.tif]
